# Systemic hypoxia has a larger effect on reducing the external load at lower exercise intensity during heart rate clamped cycling

**DOI:** 10.1002/ejsc.12204

**Published:** 2024-12-02

**Authors:** Siu Nam Li, Peter Peeling, Brendan R. Scott, Jeremiah J. Peiffer, Alex Shaykevich, Olivier Girard

**Affiliations:** ^1^ School of Human Sciences (Exercise and Sports Science) The University of Western Australia Perth Western Australia Australia; ^2^ Department of Sport Science Western Australian Institute of Sport Mount Claremont Western Australia Australia; ^3^ Physical Activity, Sport and Exercise (PHASE) Research Group School of Allied Health (Exercise Science) Murdoch University Perth Western Australia Australia; ^4^ Centre for Healthy Ageing Murdoch University Perth Western Australia Australia

**Keywords:** cycling, exercise intensity, external load, heart rate clamp, hypoxia, internal load

## Abstract

The effects of acute hypoxic exposure on mechanical output and internal responses during cycling with heart rate (HR) clamped at lactate thresholds 1 and 2 (LT1 and LT2, respectively) were investigated. On separate days, 12 trained males cycled for 15 min at a clamped HR corresponding to LT1 and LT2 under normoxic or hypoxic conditions (simulated altitude of ∼3500 m and inspired oxygen fraction of 13.6%). Power output (PO), arterial oxygen saturation, ventilatory and perceptual responses were measured every 3 min, with metabolic response assessed pre‐ and post‐exercise. At LT1, PO was consistently lower in hypoxia compared to normoxia (*p* < 0.01). At LT2, PO was not different between normoxia and hypoxia at 3 and 6 min (both *p* > 0.42) but was significantly lower in hypoxia at 9, 12 and 15 min (all *p* < 0.04). Overall, hypoxia induced a greater decrease in PO at LT1 (−33.3% ± 11.3%) than at LT2 (−18.0 ± 14.7%) compared to normoxia. Ventilatory, perceptual and metabolic responses were influenced by exercise intensity (all *p* < 0.01) but not environmental conditions (all *p* > 0.17). A simulated altitude of ∼3500 m is more effective in reducing cycling PO at LT1 than LT2 during HR clamped cycling while maintaining other internal loads. Therefore, normobaric hypoxia provides a greater benefit via a larger decrease in the mechanical constraints of exercise at lower exercise intensities.

**Highlights**

- Internal (ventilatory, metabolic and perceptual) responses remain consistent between normoxia and hypoxia (∼3500 m of simulated altitude) regardless of exercise intensity.
- Hypoxia has a greater effect on reducing the mechanical constraints of exercise when clamping heart rate (HR) at lower exercise intensities.
- The reduction in external load due to hypoxia is not immediate at the onset of exercise when HR is clamped at lactate threshold 2.

## 1 INTRODUCTION

The addition of hypoxia to exercise alters the interplay between internal and external load metrics compared to normoxic conditions (Girard et al., 2021). By clamping heart rate (HR) during cycling, normobaric hypoxia can maintain physiological stimulus with lower mechanical work compared to normoxic conditions. For instance, in trained males cycling for 60 min at simulated altitudes of 2500–4000 m, with HR clamped at 80% of the first ventilatory threshold (VT1), cycling power output (PO) decreased by ∼12.3% for every 500 m increment in simulated altitude, whereas metabolic, ventilatory, neuromuscular and perceptual responses are sustained up to ∼3500 m (Li et al., 2023a). Consequently, repeating these HR clamped sessions in normobaric hypoxia may induce similar chronic adaptations despite lower workloads compared to normoxia. For example, exercising at comparable HR (corresponding to 65% of maximal oxygen uptake [V̇O_2max_]) for 1 h per day, 3 days per week and over 4 weeks led to similar time‐to‐exhaustion improvements in overweight and obese individuals, despite a 17.5% reduction in cycling PO in hypoxia (∼2700 m) compared to normoxia (Wiesner et al., 2010). Accordingly, preserving HR between normoxia and hypoxia can lead to comparable training outcomes with less mechanical work done in hypoxia. With previous investigations primarily focusing on low‐to‐moderate exercise intensity (Li et al., 2023a; Wiesner et al., 2010), it remains unknown whether the effects of hypoxia on reducing the mechanical load persist at higher exercise intensities (i.e., at or above VT1). As such, normobaric hypoxia may also be able to reduce the mechanical load during sessions where higher exercise intensities are performed (e.g., interval sessions).

The effects of hypoxia on physiological responses vary with absolute exercise intensity, with more pronounced changes in internal loads observed when lower external workloads are sustained. For example, in normobaric hypoxia at ∼4000 m, cycling at 44% ± 5.3% of V̇O_2max_ (100 W) increased HR by ∼30%, whereas at 63% ± 7.1% of V̇O_2max_ (150 W), the increase was only ∼15% compared to normoxia after ∼10 min of exercise (Stenberg et al., 1966). Moreover, in a study involving three sets (9 × 4‐s) of repeated sprints, no differences in mean and peak PO were observed between normoxia and simulated altitudes up to ∼3000 m (Goods et al., 2014). Of note, the cohorts in these previous works (Goods et al., 2014; Stenberg et al., 1966) had varying fitness levels, which are known to influence the responses to acute hypoxic exposure (Macinnis et al., 2015). These fitness differences may have contributed to the discrepant findings in these studies. These findings suggest that the hypoxia effects on the internal load may diminish with greater reliance on anaerobic pathways due to increased external work output (Scott et al., 2016). However, research on the effects of hypoxia on sustained efforts at exercise intensities between low‐to‐moderate (<60% V̇O_2max_) and maximal (repeated sprints) domains is limited. Therefore, it is unknown whether the effects of hypoxia are altered when HR is clamped at higher submaximal exercise intensities between low‐to‐moderate and maximal. Moreover, although previous works suggest that the effects of hypoxia decrease as exercise intensity increases, the magnitude of this difference between exercise intensities is unknown.

To standardise prescribed exercise intensities across individuals with varying fitness levels, physiological landmarks are often used to induce similar relative internal strain. Lactate thresholds (LTs), determined from graded exercise tests, serve as common physiological landmarks to prescribe exercise intensity for endurance sports (Faude et al., 2009; Wolpern et al., 2015). For instance, a five‐zone model was developed for cross‐country skiers, using LT1 and LT2 to quantify exercise intensity over 60 training sessions (Seiler & Kjerland, 2006). Therefore, providing practitioners with insights into the effects of using hypoxia to reduce the mechanical load when exercise is conducted using a HR clamp at LT1 and LT2 compared to normoxia would be beneficial.

This study aimed to determine the effects of acute hypoxic exposure (∼3500 m simulated altitude) on mechanical output (cycling PO) and internal responses (i.e., arterial oxygen saturation [S_p_O_2_], metabolic, ventilatory and perceptual responses) when cycling at HR clamped at LT1 and LT2. Previous works (Stenberg et al., 1966) suggest that both LT1 and LT2 experience a decrease in cycling PO due to hypoxia. However, since exercising at LT2 requires a greater contribution of the anaerobic pathways, which are not affected by hypoxia (Scott et al., 2016), the reduction in mechanical output when HR is clamped at LT2 is likely to be lower than at LT1. Therefore, we hypothesised that, compared to LT1, the reduction in cycling PO in hypoxia *versus* normoxia would be lowered at LT2, whereas the hypoxic effect on other internal responses would not differ between the two intensities.

## 2 METHODS

### 2.1 Participants

Twelve males (training ∼8 h per week) were recruited to participate in this study (age 23.1 ± 0.4 years; stature 178.8 ± 7.0 cm; body mass 75.3 ± 7.7 kg and V̇O_2max_ 48.5 ± 3.8 mL min^−1^ kg^−1^). Participants were classified as ‘Trained/Development’ (Tier 2) using established criteria (McKay et al., 2021). A power calculation for repeated measures analysis of variance (*α* = 0.05 and 1 − *β* = 0.95) was conducted using G*POWER (version 3.1.9.3), indicating a sample size of 10 for our primary variable: cycling PO. The effect size (Cohen's *f*) was 1.73, with a critical F‐value of 4.74 derived from previous work (Amann & Dempsey, 2008). All participants were free of injury and medical conditions and resided in Perth, Western Australia (near sea‐level) for at least 6 months preceding the investigation. The study was approved by the Human Research Ethics Committee at The University of Western Australia (ROAP2021/ET000853) and was conducted in accordance with the Declaration of Helsinki. Signed written consent was obtained from the participants before inclusion.

### 2.2 Experimental protocol

Participants underwent a baseline testing and familiarisation session to determine their HR corresponding to LT1 and LT2 from blood lactate (BLa) measurements during an incremental cycling test. The test involved a stepwise increase in cycling PO by 25 W every 4 min commencing at 100 W. Both LT1 and LT2 were determined using the Dmax method (Buckley et al., 2003). Briefly, LT1 was determined as the first workload preceding a 0.4 mmol L^−1^ rise in BLa above baseline. LT2 was calculated as the workload corresponding to the greatest perpendicular distance from a regression curve relating workload to BLa and a straight line connecting the first and last points of the curve. Subsequently, participants completed four experimental trials in a randomised order, cycling for 15 min at HR clamped at LT1 or LT2 (corresponding to 56.5% ± 5.8% and 75.8% ± 5.9% of V̇O_2max_, respectively), in either normoxia or hypoxia (∼3500 m simulated altitude, F_i_O_2_ = 13.6%). All participants were blinded to the experimental conditions. Trials were separated by a minimum of 48 h and a maximum of 7 days; trials were performed at a similar time of day (±2 h) in temperate conditions (temperature 23.2 ± 0.4°C and relative humidity 49.7% ± 6.2%).

Each experimental session began with a standardised warm‐up, including 10 min of cycling at 100 W, followed by 5 min of passive rest before completing 15 min of cycling in the designated experimental condition. During the 15 min cycling bout, participants inhaled either normoxic or hypoxic (simulated altitude of ∼3500 m and inspired oxygen fraction of 13.6%) air for the entire bout. Normobaric hypoxia was induced using a hypoxic generator (F10 altitude generator, Box Altitude), which was connected to participants via a facemask at the onset of exercise, totalling 15 min of hypoxic exposure per trial. All cycling was performed on an electronically braked cycle ergometer (Wahoo kickR power trainer V5, Wahoo Fitness Inc.), with individual set‐up replicated for all trials.

### 2.3 Automatic heart rate clamp

During the 15 min cycling bout, cycling PO was continuously controlled by a custom‐made application (AutoHR) installed on an Apple iPod Touch (Apple Inc.) which has previously been shown to have an accuracy of ±2 bpm (Li et al., 2023b). Briefly, AutoHR controlled the resistance of the cycle ergometer (Wahoo kickR power trainer v5, Wahoo Fitness Inc.) in 5 s intervals via a standardised Bluetooth low‐energy profile. This control was based on target HR (either LT1 or LT2) and actual HR measurements obtained from a chest strap monitor (Polar H10).

### 2.4 Exercise‐related responses

During exercise, target and actual HR along with instantaneous cycling PO were recorded via AutoHR at 1 Hz for subsequent export and analysis. Expired gases were collected continuously using an indirect calorimetry system (TrueOne 2400, Parvo Medics Inc.), providing rolling 15 s averages of V̇O_2_, expired carbon dioxide (V̇CO_2_) and minute ventilation (V̇_E_) over the entire 15 min cycling bout. The S_p_O_2_ was estimated every 3 min via fingertip pulse oximetry (Palmsat 2500, Nonin Medical Inc.). Capillary blood samples were taken from the earlobe and analysed for BLa using a point‐of‐care analyser (Lactate Plus, Nova Biomedical) on arrival to the laboratory, after the warm‐up and immediately after the 15 min cycling bout.

Perceptual responses, including ratings of perceived exertion (RPE), sense of effort, lower‐limb discomfort and difficulty breathing, were recorded every 3 min during exercise (aligned with S_p_O_2_ measurements) using modified Borg CR10 scales (Christian et al., 2014). Briefly, the ‘sense of effort’ was assessed from the question: ‘*How hard are you trying?*’ (i.e., with the anchor points provided ranging from 0 or ‘no effort’ to 10 or ‘maximum effort’). RPE, perceived lower‐limb discomfort, and difficulty breathing were assessed from the questions: ‘*What is your overall perceived exertion?*’, ‘*How heavy do your legs feel?*’ and ‘*How difficult does it feel to breathe?*’, respectively. All questions were printed above the modified Borg CR10 scales and visible to participants at times of measurements.

### 2.5 Data processing

For cycling PO, mean values were calculated for each 3 min during the 15 min of cycling presented as 3, 6, 9, 12 and 15 min timepoints. Mean values for the last 15 min of the cycling bouts were calculated for HR, S_p_O_2_, all expired gases and perceptual responses. The difference in BLa between pre‐ and post‐cycling was used to calculate ΔBLa.

### 2.6 Data analysis

A general linear mixed model using the R (Team RC. R Core Team, 2020) libraries *MASS* and *lme4* was used to analyse the data. A Shapiro–Wilk test assessed normality. For normally distributed variables, a random intercept was included to adjust for baseline levels and inter‐individual homogeneity. All models (cycling PO: environmental condition, exercise intensity, time and environmental condition × exercise intensity × time and all other variables: environmental condition, exercise intensity and environmental condition × exercise intensity) were estimated using the restricted maximum likelihood. The *p*‐values were obtained using Type II Wald *F* tests with Kenward–Roger degrees of freedom as implemented in the R package *car*. Post hoc pairwise‐comparisons using the Tukey honestly significant difference test were performed using the R package *emmeans* if a significant main effect was observed. For non‐parametric variables, a Kruskal–Wallis test was used for multi‐condition comparisons, followed by a post hoc Dunn's test with Bonferroni correction if a significant main effect was observed. For all analyses, the accepted error rate was *α* = 0.05. Partial eta‐squared ($\eta_{p}^{2}$), with $\eta_{p}^{2}$ ≥ 0.06 representing a *moderate* effect and $\eta_{p}^{2}$ ≥ 0.14 a *large* effect, were used to calculate effect sizes (Cohen, 2013). All data are expressed as mean ± SD.

## 3 RESULTS

### 3.1 Power output

**FIGURE 1:**
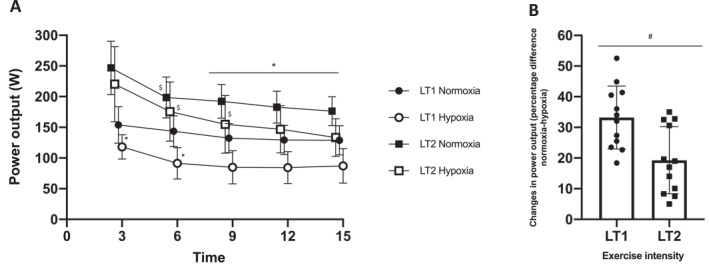
Power output (PO) (A) during the 15 min cycling bout at each condition and % difference in PO (B) between normoxia and hypoxia at heart rate (HR) clamped at lactate threshold 1 and lactate threshold 2. Values are means ± SD (*n* = 12). LT1, HR clamped at lactate threshold 1 and LT2, HR clamped at lactate threshold 2. ^$^Significant difference compared to previous timepoint. *Significant difference between normoxia and hypoxia. ^#^Significant difference between LT1 and LT2.

An interaction effect was detected for cycling PO (*p* < 0.01, Figure 1A). Post hoc analysis revealed that, at LT1, cycling PO was lower in hypoxia compared to normoxia at every timepoint (*p* < 0.01). There was no effect of time for both LT1 normoxia and LT1 hypoxia (*p* > 0.05, Figure 1A). At LT2, cycling PO in both normoxia and hypoxia was lower at 6 min compared to 3 min (both *p* < 0.01), with no difference between conditions (both *p* > 0.42, Figure 1A). Cycling PO at LT2 hypoxia further decreased at 9 min and was significantly lower than LT2 normoxia at 9, 12 and 15 min timepoints (all *p* < 0.04, Figure 1A). The reduction of cycling PO relative to 3 min was not different between 6, 9 and 12 min (all *p* > 0.41), whereas the decrease in cycling PO was significantly lower at 15 min compared to all other timepoints (all *p* < 0.01, Figure 1A).

Between environmental conditions, a main effect was observed, where the addition of hypoxia reduced cycling PO by 33.3% ± 11.3% when HR was clamped at LT1, which was significantly greater than the reduction of 18.0% ± 14.7% observed when HR was clamped at LT2 (*p* = 0.02, Figure 1B).

A main effect for exercise intensity was present (*p* < 0.01, Figure 1A). Post hoc analysis revealed that compared to LT1, cycling PO was significantly greater at LT2 by 36.6% ± 14.4% in normoxia and 55.7% ± 20.5% in hypoxia, with the difference in hypoxia was significantly greater than in normoxia (*p* = 0.03).

### 3.2 Metabolic response

No interaction effect was detected for ΔBLa (*p* = 0.81, Table 1).

**TABLE 1:** Mean indices of the internal load during the 15 min of heart rate clamped cycling.

Variable	Exercise intensity	Environmental condition		Environment × intensity		Environment		Intensity	
		Normoxia	Hypoxia	*F* value	*p*‐value ($\eta_{p}^{2}$)	*F* value	*p*‐value ($\eta_{p}^{2}$)	*F* value	*p*‐value ($\eta_{p}^{2}$)
Heart rate (bpm)	LT1	136.9 ± 9.7	137.5 ± 9.3	0.07	0.87 (*<0.01*)	0.01	0.95 (*<0.01*)	253.90	**<0.01** (*0.90*)
	LT2	163.1 ± 6.9^a^ ^,^ ^b^	162.9 ± 7.0^a^ ^,^ ^b^						
Arterial oxygen saturation (%)	LT1	95.2 ± 0.8	77.8 ± 4.3^a^ ^,^ ^c^	0.25	0.62 (*<0.01*)	609.60	**<0.01** (*0.16*)	4.47	0.27 (*0.06*)
	LT2	94.1 ± 1.1	75.9 ± 2.2^a^ ^,^ ^c^						
Δ blood lactate concentration (mmol L^−1^)	LT1	0.9 ± 0.8	1.1 ± 0.9	0.10	0.81 (*<0.01*)	0.78	0.50 (*0.01*)	99.00	**<0.01** (*0.57*)
	LT2	5.5 ± 2.4	6.1 ± 3.4						
Oxygen consumption (ml min^−1^ kg^−1^)	LT1	26.7 ± 4.2	25.5 ± 5.2	0.49	0.64 (*<0.01*)	0.04	0.89 (*<0.01*)	61.90	**<0.01** (*0.67*)
	LT2	36.8 ± 4.7^a^ ^,^ ^b^	37.5 ± 11.0^a^ ^,^ ^b^						
Carbon dioxide expired (L min^−1^)	LT1	1.86 ± 0.3	1.55 ± 0.3	0.20	0.79 (*<0.01*)	29.59	0.17 (*0.21*)	167.30	**<0.01** (*0.85*)
	LT2	2.77 ± 0.3^a^ ^,^ ^b^	2.37 ± 0.5^a^ ^,^ ^b^						
Minute ventilation (L min^−1^)	LT1	50.3 ± 9	51.2 ± 9.9	0.43	0.68 (*<0.01*)	1.06	0.52 (*0.01*)	189.0	**<0.01** (*0.86*)
	LT2	82.9 ± 12.0^a^ ^,^ ^b^	87.1 ± 19.1^a^ ^,^ ^b^						
Rating of perceived exertion (0–10 au)	LT1	2.8 ± 0.9	2.3 ± 0.9	0.83	0.37 (*0.02*)	0.55	0.46 (*0.02*)	81.10	**<0.01** (*0.71*)
	LT2	5.2 ± 1.4^a^ ^,^ ^b^	5.2 ± 1.9^a^ ^,^ ^b^						
Sense of effort (0–10 au)	LT1	2.7 ± 1	2.2 ± 1	0.36	0.55 (*0.02*)	0.65	0.43 (0.01)	68.40	**<0.01** (*0.67*)
	LT2	5.2 ± 1.6^a^ ^,^ ^b^	5.1 ± 2.0^a^ ^,^ ^b^						
Leg discomfort (0–10 au)	LT1	2.3 ± 1.3	1.9 ± 1.2	0.70	0.41 (*0.02*)	0.12	0.73 (*<0.01*)	60.90	**<0.01** (*0.65*)
	LT2	4.6 ± 1.7^a^ ^,^ ^b^	4.8 ± 2.0^a^ ^,^ ^b^						
Difficulty breathing (0–10 au)	LT1	2.3 ± 1.1	2.7 ± 1.1	3.00	0.09 (*0.08*)	9.40	0.34 (*0.22*)	37.20	**<0.01** (*0.53*)
	LT2	3.5 ± 1.5^a^ ^,^ ^b^	3.8 ± 2.0^a^ ^,^ ^b^						

*Note*: Data are presented as mean ± SD. Significant *p‐*values (<0.05) are highlighted in bold. Italic values denote partial eta squared ($\eta_{p}^{2}$) results in parentheses.

Abbreviations: LT1, heart rate clamped at lactate threshold 1 and LT2, heart rate clamped at lactate threshold 2.

^a^ Significant difference compared to LT1 normoxia.

^b^ Significant difference compared to LT1 hypoxia.

^c^ Significant difference compared to LT2 normoxia.

There was no effect of environmental condition on ΔBLa (*p* = 0.50, Table 1). The ΔBLa was significantly affected by exercise intensity (*p* < 0.01, Table 1), with the post hoc analysis revealing significantly greater ΔBLa at LT2 compared to LT1 (*p* < 0.01).

### 3.3 Ventilatory responses

No interaction effects were present for all ventilatory responses (all *p* > 0.64, Table 1).

There was no effect of environmental condition on V̇O_2_, V̇CO_2_ and V̇_E_ (all *p* > 0.17, Table 1), whereas exercise intensity had a significant effect on these variables (all *p* < 0.01, Table 1). Post hoc analysis showed higher values when HR was clamped at LT2 compared to LT1 (all *p* < 0.01, Table 1).

### 3.4 Perceptual responses

No interaction effects were observed for all perceptual responses (all *p* > 0.54, Table 1).

No effect of environmental condition was noted for all perceptual responses (all *p* > 0.27, Table 1).

There was an effect of exercise intensity on perceptual responses (all *p* < 0.01, Table 1), with RPE, sense of effort, leg discomfort and difficulty breathing being elevated at LT2 compared to LT1 (all *p* < 0.01, Table 1).

### 3.5 Heart rate

No interaction effect was noted for HR (*p* = 0.87, Table 1).

There was no effect of environmental condition on HR (*p* = 0.95). Overall, HR was greater at LT2 in both normoxia and hypoxia compared to LT1 in the same environmental conditions (*p* < 0.01, Table 1).

### 3.6 Arterial oxygen saturation

No interaction effect was present for S_p_O_2_ (*p* = 0.62, Table 1).

There was an effect of environmental condition of S_p_O_2_, with lower values in both LT1 and LT2 trials in hypoxia compared to normoxia (*p* < 0.01, Table 1). There was no effect of exercise intensity on S_p_O_2_ (*p* = 0.27, Table 1).

## 4 DISCUSSION

We examined the acute effects of hypoxic exposure on mechanical output and internal responses during cycling with HR clamped at LT1 and LT2. Our main findings indicate that (1) internal load metrics (ventilatory, metabolic and perceptual responses) remain consistent across environmental conditions regardless of exercise intensity; (2) hypoxia has a greater effect on reducing cycling PO when HR is clamped at LT1 compared to LT2 and (3) when HR is clamped at LT2, the effects of hypoxia on cycling PO are not apparent until 9 min after the onset of exercise compared to normoxia. These findings collectively support our initial hypothesis that, compared to LT1, the reduction in cycling in hypoxia versus normoxia would be less pronounced at LT2, whereas the hypoxic effect on other internal responses would not differ between the two intensities.

### 4.1 Power output

This study is the first to compare the effects of hypoxia on cycling PO when clamping HR at LT1 and LT2. When using hypoxia to reduce mechanical output while maintaining internal load metrics, clamping HR at LT1 resulted in greater decrease in cycling PO compared to LT2 (−33.3% and −14.7%, respectively). These reductions in cycling PO at LT1 are consistent with previous works when HR was clamped at sub‐threshold exercise intensities. For example, Li et al. (2023a) showed similar decreases (∼34%) in cycling PO after 15 min of cycling at ∼3500 m when HR was clamped at 80% of VT1. Our results also showed that the hypoxic‐induced decrease in cycling PO was significantly greater at LT1 and LT2. This discrepancy is likely due to the effect of exercise intensity on the relative contribution between aerobic and anaerobic energy pathways. Previous research has shown that during incremental exercise tests, the relative contribution of anaerobic energy systems gradually increases with progressive workload increments (Bertuzzi et al., 2013). Therefore, the relative contribution of anaerobic energy systems is greater when HR is clamped at LT2 compared to LT1 as evidenced by the greater accumulation of BLa when HR was clamped at LT2. Additionally, it has been established that maximal intensity exercises, where the relative anaerobic contribution is substantial (i.e., single sprint exercises), are not affected by hypoxia (Girard et al., 2017). Therefore, the lesser decrement in cycling PO observed at LT2 compared to LT1 is likely due to the increased anaerobic energy contribution at higher exercise intensity. Overall, our results show that hypoxia effectively reduce cycling PO when HR is clamped at LT1 and LT2, with a more prominent effect at LT1 than LT2.

Interestingly, hypoxia did not manifest its effects until 9 min after HR was clamped at LT2, likely due to differences in energy system contributions compared to LT1. For example, during all‐out exercise testing, the contribution of anaerobic energy systems (i.e., glycolysis) gradually decreases as exercise duration increases (Gastin, 2001). Therefore, in our results where HR was clamped, the effects of hypoxia to reduce cycling PO may have been compensated for at the onset of exercise through glycolysis, resulting in no difference in cycling PO between normoxia and hypoxia. Subsequently, as the relative contribution of aerobic energy systems increased, the decrease in cycling PO was observed in LT2. Our findings imply that when hypoxia is employed to reduce mechanical output during exercise at HR corresponding to LT2, any reduction may go unnoticed unless the exercise duration exceeds 9 min. Practically, as exercise intensities around LT2 are commonly used to prescribe interval training sessions, using hypoxia to lessen the mechanical load may not be suitable for all interval training sessions. For example, work durations at LT2 have been previously described as 4 × 4 min, 4 × 8 min and 4 × 16 min, representing short, medium and long intervals, respectively (Seiler et al., 2013). In this context, only long intervals would exhibit a decrease in the mechanical load due to hypoxia exposure, whereas no difference in cycling PO would be observed during medium and short intervals. Consequently, clamping HR at LT2 is limited during work intervals shorter than 9 min. Overall, our findings suggest that when clamping HR at LT2, the duration of exercise needs to exceed 9 min for hypoxia to reduce the external load.

### 4.2 Internal load response

Our study is the first to investigate whether the effects of hypoxia on the internal load responses during HR clamped cycling persist across LT1 and LT2. Our findings indicate that, when clamping HR at LT1 and LT2, only exercise intensity, and not hypoxic exposure, had an effect on metabolic, perceptual and ventilatory responses. This is likely due to the reduction in cycling PO observed at each intensity, which aligns with previous findings. For instance, when clamping HR at 80% of VT1, a simulated altitude of ∼3500 m did not affect BLa or perceptual responses compared to normoxia during cycling for 60 min (Li et al., 2023a). Additionally, substantial evidence has shown that when performing the same amount of work, hypoxia exacerbates ventilatory responses. For example, during fixed submaximal work rates of ∼47.2% and 77.3% of V̇O_2max_ (73 and 146 W, respectively), exposure to ∼3000 m of simulated altitude elevated internal exercise loads at both exercise intensities indicated by higher values of V̇O_2_ (+9.7% and +7.9%, respectively), V̇_E_ (+19.5% and +26.3%, respectively) and HR (+7.8% and +5.2%, respectively) (Kapus et al., 2017). In our study, where HR was clamped, metabolic, perceptual and ventilatory responses would have remained consistent between normoxia and hypoxia through a reduction in external work regardless of whether HR was clamped at LT1 or LT2. Therefore, the internal load of exercise is only affected by exercise intensity, and not environmental condition, when performing HR clamped cycling.

## 5 LIMITATIONS

It has previously been outlined that various factors can contribute to an individual's response to acute hypoxic exposure (Soo et al., 2020). Our results demonstrate this individual variation through the relative decreases in cycling PO due to hypoxia at each exercise intensity. A possible explanation for this variation could be the training status of tested individuals. For instance, incremental testing results revealed that trained subjects had a greater decrement in V̇O_2max_ due to hypoxia compared to untrained individuals (Mollard et al., 2007). Therefore, it remains unknown whether our results derived from trained males are replicable on other cohorts (i.e., clinical populations studied in Wiesner et al., 2010) with significantly different aerobic fitness levels. Nevertheless, our results provide a basis for understanding the effects of hypoxia on internal and external load metrics when HR is clamped at LT1 and LT2.

### 5.1 Practical implications

Across exercise intensities (HR corresponding to LT1 and LT2), hypoxia can effectively reduce the mechanical load of exercise. This provides a valuable tool for coaches and practitioners to lessen the mechanical burden of exercise for load compromised individuals (e.g., athletes during periods of high training load and clinical populations suffering musculoskeletal conditions) while maintaining the cardiometabolic solicitation of exercise compared to normoxia (Girard et al., 2021). However, practitioners should be aware that the effects of hypoxia are greater at lower compared to higher exercise intensities and careful consideration may be required regarding the duration of cycling bout should hypoxia be applied to interval training sessions to reduce the mechanical load. Nevertheless, hypoxia can be effectively used in combination with a HR clamp to reduce the external load of exercise while maintaining a comparable internal load with normoxia.

## 6 CONCLUSION

When clamping HR at LT1 and LT2, metabolic, ventilatory and perceptual responses are maintained despite a reduction in cycling PO in hypoxia (∼3500 m of simulated altitude) compared to normoxia at both exercise intensities. However, the impact of hypoxia on reducing mechanical work is greater at LT1 than at LT2 as evidenced by the relative decrease in cycling PO. Additionally, the reduction in mechanical output at LT2 due to hypoxia is not apparent until 9 min after the onset of exercise. Therefore, while hypoxia can reduce cycling PO at both LT1 and LT2, it likely provides greater benefit at the lower intensity, where a more pronounced decrease in cycling PO can be achieved for similar internal load responses compared to normoxia.

## CONFLICT OF INTEREST STATEMENT

The authors declare that they have no conflicts of interest.
